# Impact toughness and dynamic constitutive model of geopolymer concrete after water saturation

**DOI:** 10.1038/s41598-024-57760-1

**Published:** 2024-03-26

**Authors:** Tiecheng Yan, Xiangxiang Yin, Xingyuan Zhang

**Affiliations:** 1 College of Civil Engineering, Longdong University, Qingyang, 745000 China; 2 Hydrochina Chengdu Engineering Corporation Limited, Chengdu, 610072 China; 3 College of Water Conservancy and Hydropower, Hohai University, Nanjing, 210098 China

**Keywords:** Geopolymer concrete, Water saturation, Impact toughness, Constitutive model, Statistical damage distribution theory, Engineering, Materials science

## Abstract

The dynamic compression test of geopolymer concrete (GC) before and after water saturation was carried out by the split Hopkinson pressure bar (SHPB). And the effects of water saturation and strain rate on impact toughness of GC were studied. Based on Weibull statistical damage distribution theory, the dynamic constitutive model of GC after water saturation was constructed. The results show that the dynamic peak strain and specific energy absorption of GC have strain rate strengthening effect before or after water saturation. The impact toughness of GC decreases after water saturation. The size distribution of GC fragments has fractal characteristics, and the fractal dimension of GC fragments after water saturation is smaller than that before water saturation. The dynamic constitutive model based on Weibull statistical damage distribution theory can accurately describe the impact mechanical behavior of GC after water saturation, and the model fitting curves are in good agreement with the experimental stress–strain curves.

## Introduction

Geopolymer concrete (GC) is a new building material. Because of its low energy consumption, low carbon dioxide emissions, and large consumption of industrial solid waste in the production process, GC is reputed as the “green and environment-friendly material” in the twenty-first century^1–3^. Researches have shown that GC not only has high strength and good workability, but also has excellent durability, which makes GC can be used in hydraulic engineering and has a wide application prospects in hydraulic structures (such as dams, locks, docks and bridges)^4–6^.

There are many tiny pores inside the concrete. When serving in the water environment, its internal pores are saturated or semi-saturated, which will affect the mechanical properties of concrete. Many researches have shown that the mechanical properties of concrete after water saturation are quite different from those at the dry state^7,8^. At present, the researches on the mechanical properties of concrete after water saturation are mainly limited to quasi-static cases, and there are still some deficiencies among the researches of its dynamic mechanical properties^9–11^. Rossi et al. studied the effect of free water on the properties of concrete under tensile stress, and explained the change mechanism of mechanical properties of concrete after water saturation. But they did not study the compressive strength of concrete^12,13^. Mehta et al. and Vu et al. studied the strength characteristics of concrete under various moisture content states, but did not deeply explore the mechanism of free water^14,15^. Zhang et al. compared the change law of compressive strength of concrete after water saturation under different loading rates, but the loading rates were less than 10^–4^ s^−1^, which still belonged to the quasi-static loading state^16^.

As a rate-sensitive material, concrete will show different mechanical properties under impact load and static load^17–20^. And in the actual service environment, hydraulic concrete structures are inevitably subjected to dynamic loads such as ship strikes, underwater blasting, seismism and so on^21–24^. Therefore, it is of great significance to study the dynamic mechanical properties of concrete after water saturation for underwater blasting excavation, underwater building demolition and seismic safety assessment of large underwater buildings. Based on this, the dynamic compression test of GC after 180 d immersing in-water was carried out by the *Φ*100 mm split Hopkinson pressure bar (SHPB). And the impact toughness and fracture morphology of GC before and after water saturation were compared and analyzed. Based on the Weibull statistical damage distribution theory, the dynamic constitutive model of GC after water saturation was constructed.

## Experiment

### Preparation of specimens

The raw materials of GC include cementitious materials, alkaline activators, gravel, sand and water. Cementitious materials include slag and fly ash. Alkali activators include NaOH and sodium silicate. The purity of NaOH is more than 97%. The modulus Sodium silicate (SiO_2_/Na_2_O) is 3.1 ~ 3.4, the SiO_2_ content is more than 26.0%, and the Na_2_O content is more than 8.2%. Gravel: limestone gravel with a density of 2.70 g/cm^3^, a particle size of 5 ~ 20 mm, and a mud content of 0.2%. Sand:medium sand with a fineness modulus of 2.8, a density of 2.63 g/cm^3^, and a mud content of 1.1%. Water:clean tap water.

**Figure 1:**
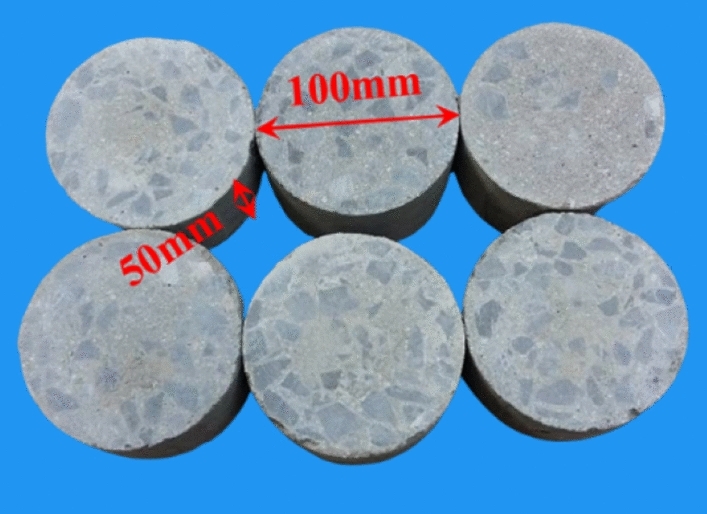
The GC specimen.

GC was prepared according to the mix proportion shown in Table 1. (1) NaOH, sodium silicate and water were mixed and stirred evenly to prepare mixture solution. (2) Sand and half of the mixture solution were added to the concrete mixer and stirred for 30 s. (3) Gravel was added to the concrete mixer and stirred for 30 s. (4) Slag and fly ash were added to the concrete mixer and stirred for 60 s. (5) The remaining mixture solution was added to the concrete mixer and stirred for 120 s. (6) Molding, vibrating and curing. The size of specimen is *Φ*100 mm × 50 mm, as shown in Fig. 1.

**Table 1:** The mix proportion of GC (kg/m^3^).

Slag	Fly ash	NaOH	Sodium silicate	Gravel	Sand	Water
249	110	25	88	1016	810	88

After standard curing of 28 d, the specimens were divided into two groups. One group of specimens (GC-D) continued standard curing for 180 d. And the other group of specimens (GC-W) were immersed in water for 180 d to make them saturated, and then they were taken out for testing.

### Experimental method

**Figure 2:**
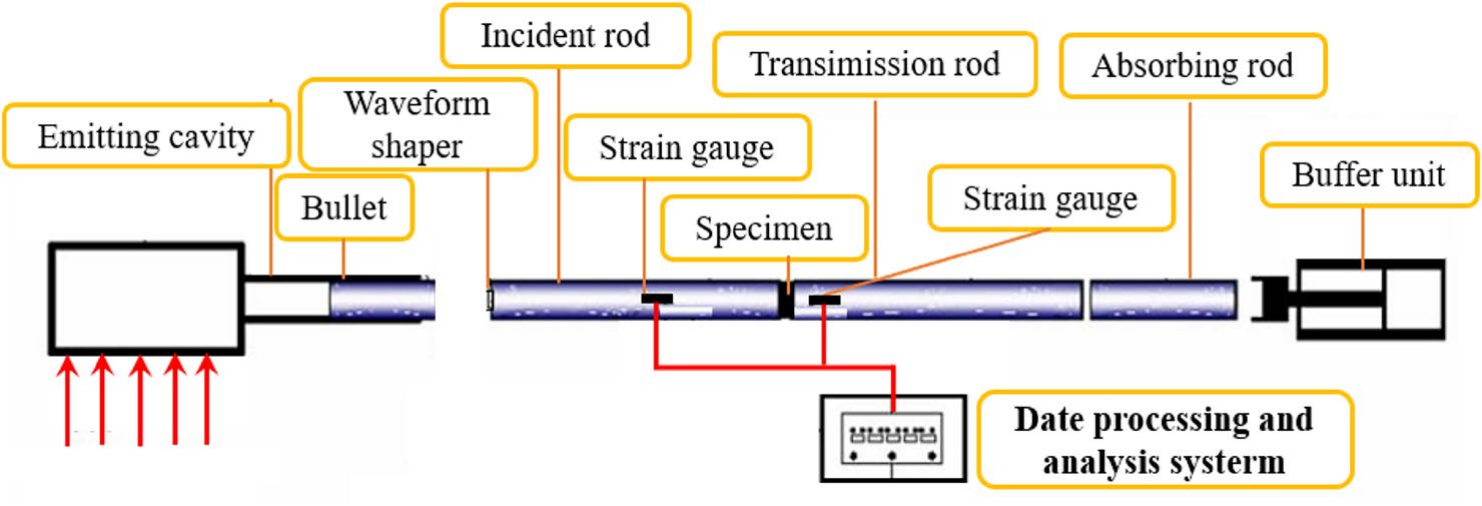
The *Φ*100 mm SHPB.

**Figure 3:**
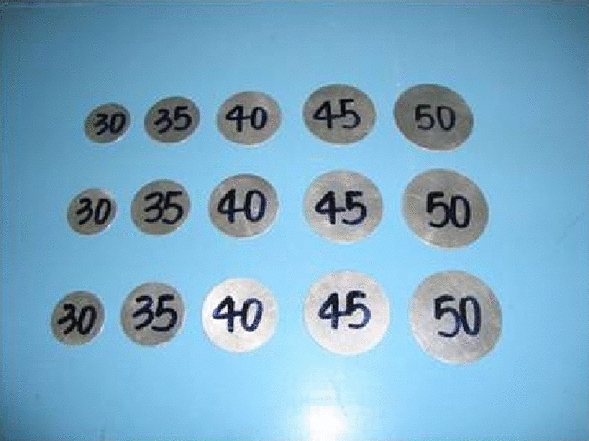
Aluminum wafers.

The dynamic compression test of GC was carried out by the *Φ*100 mm SHPB (as shown in Fig. 2). The aluminum wafers were used as the waveform shaper in the test, as shown in Fig. 3. The strains of incident, reflected and transmitted waves were measured by strain gauge, and then the test data are processed by Eq. (1), so as to obtain the stress–strain curves^25^.

1 $$ \begin{gathered} \sigma \left( t \right) = \frac{{E_{e} A_{e} }}{{2A_{s} }}\left[ {\varepsilon_{I} \left( t \right) + \varepsilon_{R} \left( t \right) + \varepsilon_{T} \left( t \right)} \right] \hfill \\ \varepsilon \left( t \right) = \frac{{C_{e} }}{L}\mathop \smallint \limits_{0}^{\tau } \left[ {\varepsilon_{I} \left( t \right) - \varepsilon_{R} \left( t \right) - \varepsilon_{T} \left( t \right)} \right]dt \hfill \\ \varepsilon^{\prime}\left( t \right) = \frac{{C_{e} }}{L}\left[ {\varepsilon_{I} \left( t \right) - \varepsilon_{R} \left( t \right) - \varepsilon_{T} \left( t \right)} \right] \hfill \\ \end{gathered} $$

where $\sigma$*,* $\varepsilon$ and $\varepsilon^{\prime}$ are the stress, strain and strain rate, respectively. $\varepsilon_{I}$, $\varepsilon_{R}$ and $\varepsilon_{T}$ are the strains of incident, reflected and transmitted waves, respectively. *L* is 50 mm. *A*_*e*_ and *A*_s_ are 7850 mm^2^. *E*_*e*_ and *C*_*e*_ are 210 GPa and 5172 m/s, respectively.

**Fig.4:**
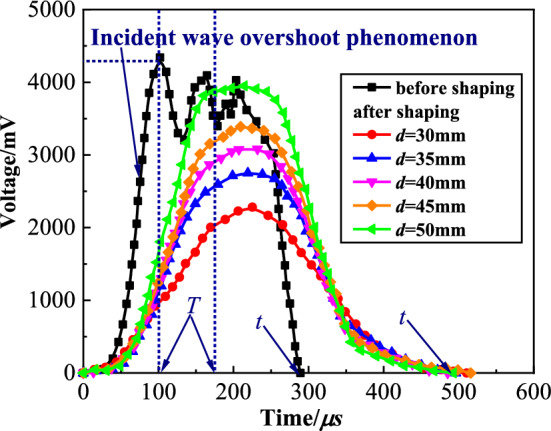
The incident wave after shaping.

The incident wave after shaping is shown in Fig. 4. From this figure, the incident wave before shaping is a trapezoidal wave, and the incident wave overshoot phenomenon is obvious. The total loading time (*T*) and time of rising segment (*t*) of incident wave are 288 μs and 101 μs, respectively. The incident wave after shaping is a half-sine wave, and the incident wave overshoot phenomenon is improved. The *T* of the incident wave increases to about 500 μs, and the *t* increases to 175 μs. The incident wave after shaping can ensure that the specimen has enough time to achieve stress uniformity before failure and achieve approximate constant strain rate loading.

**Figure 5:**
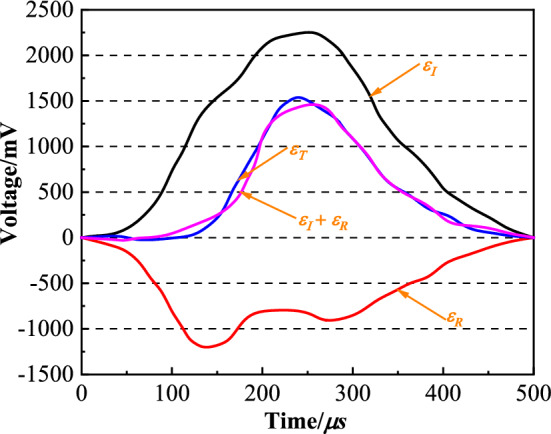
The original waves after shaping.

**Figure 6:**
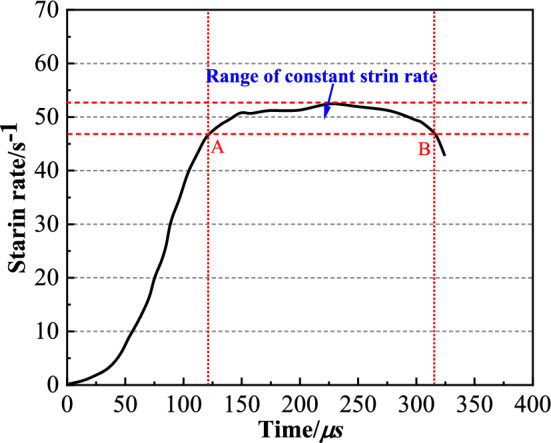
The strain rate time-course curve.

**Figure 7:**
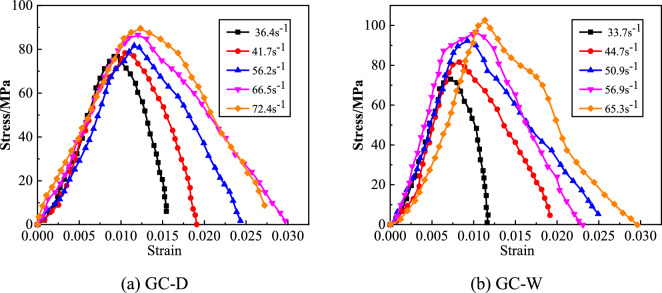
The stress–strain curves of GC.

The original waves and strain rate time-course curve after shaping are shown in Fig. 5 and Fig. 6, respectively. From Fig. 5, $\varepsilon_{I} + \varepsilon_{R} = \varepsilon_{T}$, indicating that the specimen reaches a uniform stress state. From Fig. 6, approximate constant strain rate loading was achieved during the test. The stress–strain curves obtained by the test are shown in Fig. 7.

## Results and discussion

### Impact toughness

Impact toughness is an important index to measure the dynamic mechanical properties of materials, which reflects the deformation and energy absorption capacities of materials^26^. The indexes for evaluating the impact toughness of GC are dynamic peak strain and specific energy absorption. Dynamic peak strain reflects the deformation characteristics of GC. Specific energy absorption is the area formed by the stress–strain curve and coordinate axis, which reflects the energy absorption characteristics of GC.

#### Dynamic peak strain

**Figure 8:**
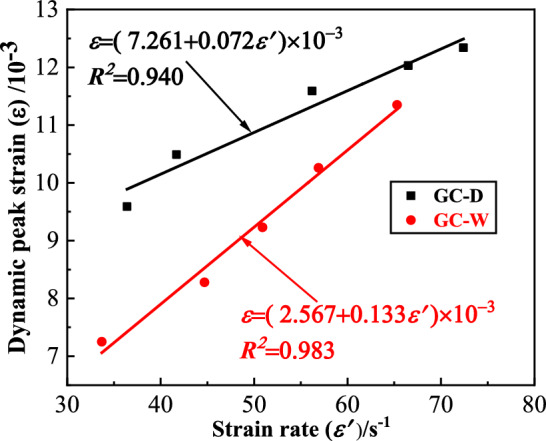
The dynamic peak strain of GC.

**Figure 9:**
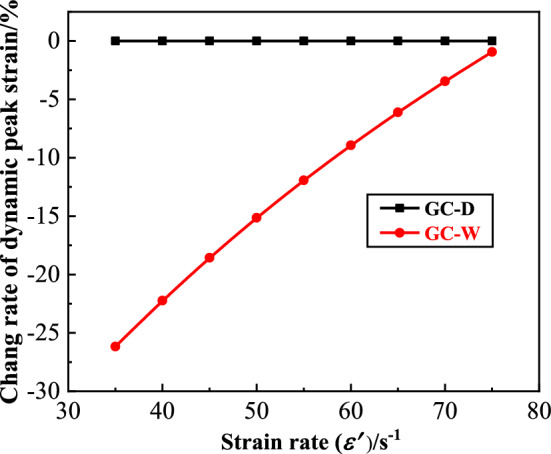
The change rate of dynamic peak strain of GC.

The dynamic peak strain ($\varepsilon$) of GC is shown in Fig. 8. From this figure, the strain rate strengthening effect of the $\varepsilon$ of GC before or after water saturation is significant. With the increase of strain rate, the $\varepsilon$ increases linearly. At the same strain rate, the $\varepsilon$ of GC before water saturation is larger than that after water saturation. In order to quantitatively analyze the influence of water saturation on the $\varepsilon$ of GC, the change rate of dynamic peak strain ($\overline{\varepsilon }$) is defined, as shown in Eq. (2).

2 $$ \overline{\varepsilon } = \left( {\varepsilon_{GC - W} - \varepsilon_{GC - D} } \right)/\varepsilon_{GC - D} $$

where $\varepsilon_{GC - W}$ is the $\varepsilon$ of GC after water saturation, $\varepsilon_{GC - D}$ is the $\varepsilon$ of GC before water saturation. The $\overline{\varepsilon }$ of GC is shown in Fig. 9. From this figure, the deformation characteristics of GC decreases after water saturation. With the increase of strain rate, the $\overline{\varepsilon }$ of GC after water saturation increases continuously. When the strain rate is small, the $\varepsilon$ of GC after water saturation decreases significantly. When the strain rate is large, the extent of the decrease of the $\varepsilon$ of GC after water saturation is small. The maximum extent of the decrease of the $\varepsilon$ of GC after water saturation is 26.16%.

It can be seen from the test results that the deformation characteristics of GC decreases after water saturation. The main reason is that the free water in pores and cracks limits the deformation of GC, which reduces its dynamic peak strain. GC is a heterogeneous material, which contains different types and sizes of pores and cracks. On the one hand, water in the pores and cracks of GC is more difficult to compress than air. Under the impact load, the volume of water is difficult to change, and the water is difficult to discharge in a short time^12,27^. Therefore, the water in the pores and cracks of GC after water saturation limits the deformation of it. On the other hand, according to the micro fluid mechanics, the viscosity of free water is greatly improved for the pores and cracks with small scale^28^. The viscosity of water applies a reverse force on the development of pores and cracks, which hinders the development of pores and cracks. With the increase of strain rate, the damage and deformation of GC is accelerated, and the ability of free water in pores and cracks to limit its deformation is relatively weakened. Therefore, the extent of the decrease of dynamic peak strain of GC declines with the increase of strain rate.

#### Specific energy absorption

**Figure 10:**
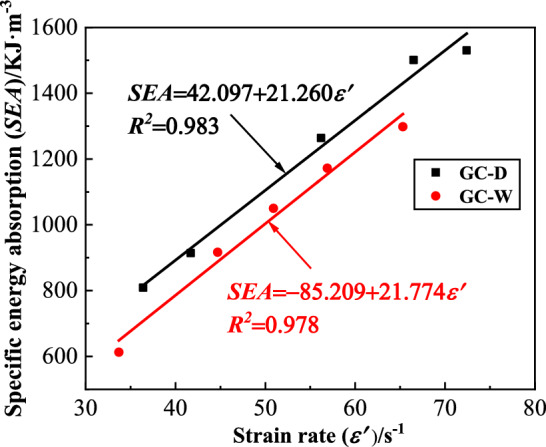
The specific energy absorption of GC.

**Figure 11:**
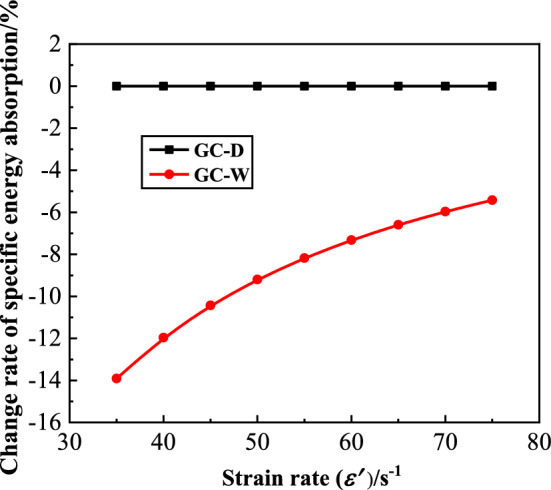
The change rate of specific energy absorption of GC.

The specific energy absorption (*SEA*) of GC is shown in Fig. 10. From this figure, with the increase of strain rate, the *SEA* of GC before or after water saturation gradually increases. At the same strain rate, the *SEA* of GC after water saturation is smaller than that before water saturation. Thus, the energy absorption characteristics of GC decreases after water saturation. In order to quantitatively analyze the influence of water saturation on the *SEA* of GC, the change rate of specific energy absorption ($\overline{SEA}$) is defined, as shown in Eq. (3).

3 $$ \overline{SEA} = \left( {SEA_{GC - W} - SEA_{GC - D} } \right)/SEA_{GC - D} $$

where $SEA_{GC - W}$ is the *SEA* of GC after water saturation, $SEA_{GC - D}$ is the *SEA* of GC before water saturation. The $\overline{SEA}$ of GC is shown in Fig. 11. From this figure, the $\overline{SEA}$ of GC after water saturation is less than 0, and the minimum is − 13.90%. With the increase of strain rate, the $\overline{SEA}$ of GC after water saturation increases. Water saturation can decrease the energy absorption characteristics of GC, but with the increase of strain rate, the extent of the decrease gradually declines.

The decrease of material surface energy due to water saturation is the main factor leading to the decrease of energy absorption characteristics of GC. According to Griffith fracture mechanics theory, the strain energy inside GC gradually increases under loading^29^. When a limit value is reached, most of the strain energy is released in the form of surface energy, resulting in the formation and expansion of cracks until the specimen is destroyed. Therefore, the formation and development of cracks need to constantly overcome the surface energy of material. According to the Young’s equation, the surface energy ($\gamma_{s1}$) at the solid–liquid surface can be expressed as Eq. (4) ^30^.

4 $$ \gamma_{s1} = \gamma_{s} - \gamma_{1} \cos \theta $$

where $\gamma_{s}$ is the surface energy of dry solid, $\gamma_{1}$ is the surface energy of saturated liquid, and $\theta$ is the contact angle between solid and liquid. From Eq. (4), when the specimen is immersed in water, the water fills the non-closed pores of the specimen, and the value of *θ* increases. Because the GC is a hydrophilic material, the value of *θ* can be approximately taken as 0 after water saturation. So the value of $\gamma_{s1}$ decreases, that is, the surface energy at the solid–liquid surface decreases. Therefore, the surface energy of the GC needed to overcome after water saturation decreases, which leads to the decrease of the energy absorption characteristics of GC after water saturation. With the increase of strain rate, the accumulation rate of strain energy and development rate of cracks increase, resulting in a relatively small change of the surface energy at the solid–liquid surface. Therefore, the extent of the decrease of energy absorption characteristics of GC after water saturation declines with the increase of strain rate.

### Fractal characteristics of fragments

**Figure 12:**
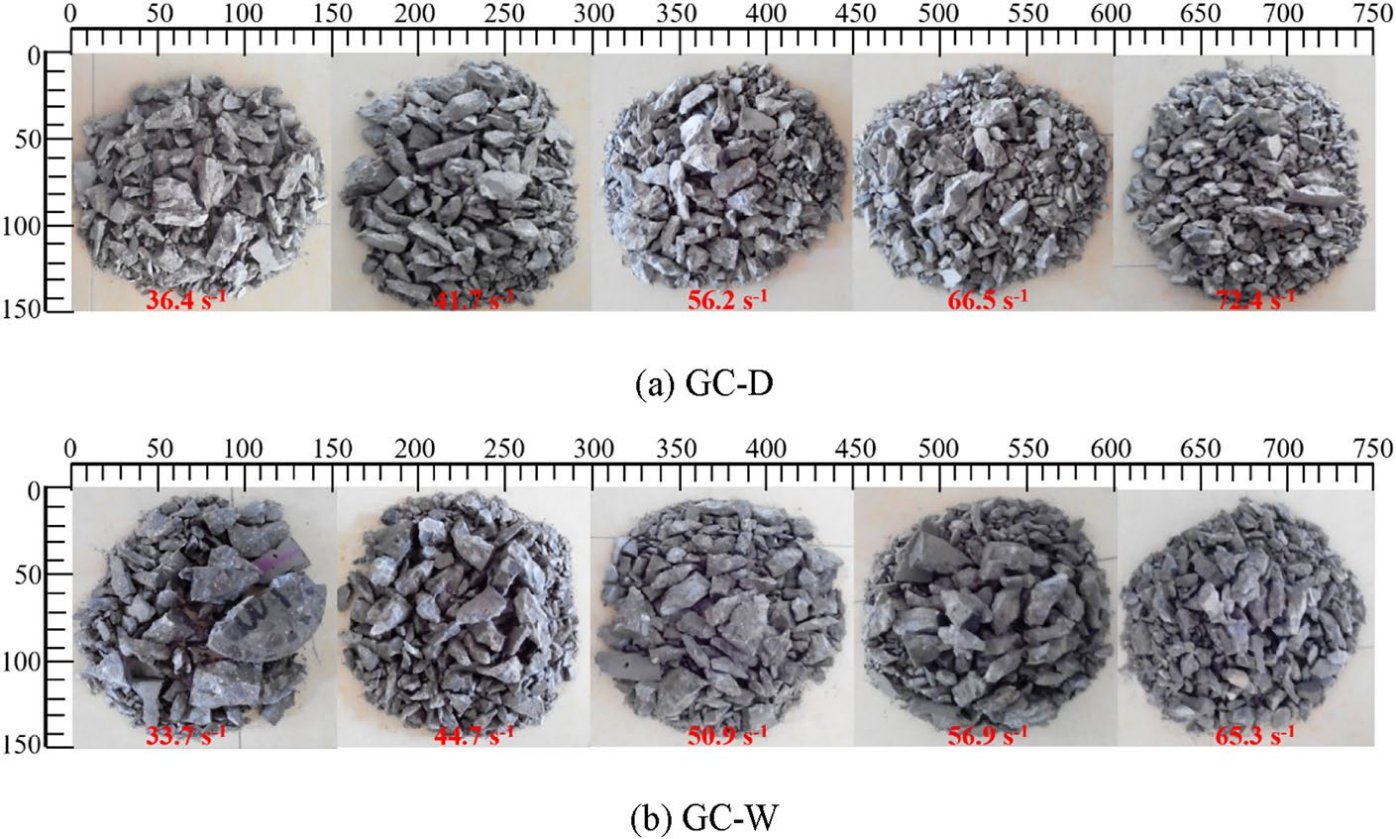
The impact fragmental morphology of GC.

The fragmental morphology and fragment size of the specimen can directly reflect the damage degree of GC. The impact fragmental morphology of GC is shown in Fig. 12. From this figure, it is difficult to judge the damage degree and internal damage of the specimen. Fractal dimension is an important concept in fractal theory to characterize the complexity and irregularity of fractal sets^31^. Due to the complexity and disorder of damage of GC under impact load, it is a typical nonlinear problem^32^. And it is difficult to use traditional theory to quantitatively describe the damage evolution behavior of GC. Therefore, the fractal dimension is used to quantify the damage degree and internal damage of the specimen.

The fragment size of concrete conforms to the G-G-S distribution^33^, and the distribution equation is shown in Eq. (5) ^34^.

5 $$ \begin{gathered} y = M\left( r \right)/M_{T} = \left( {r/r_{m} } \right)^{b} \hfill \\ N = r^{{ - D_{b} }} \hfill \\ \end{gathered} $$

where the *D*_*b*_ and $M_{T}$ are the fractal dimension and the total mass of fragments, respectively. The *r* and $r_{m}$ are the size and maximum size of fragments, respectively. the *N* and $M\left( r \right)$ are the number and cumulative mass of fragments with particle size larger than *r*, respectively.

Because it is difficult to accurately calculate the number of fragments of each particle size, the relationship between the $dN$ and the $dM$ of fragments is considered, as shown in Eq. (6).

6 $$ dM\sim r^{3} dN $$

where the $dN$ and $dM$ are the number increment and the mass increment of fragments, respectively.

The Eq. (5) are differentiated and substituted to Eq. (6), then the fractal dimension (*D*_*b*_ = 3*-b*) can be calculated. The slope of *ln[M(r)/M*_*T*_*]-lnr* curve is 3*-D*_*b*_. And there is a positive correlation among the damage degree and internal damage of specimen and fractal dimension.

**Figure 13:**
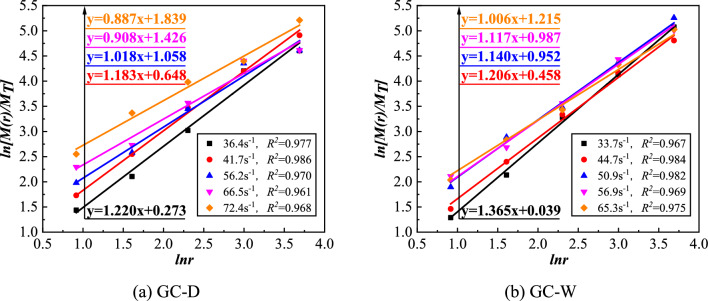
The *ln[M(r)/M*_*T*_*]-lnr* curves of GC.

**Figure 14:**
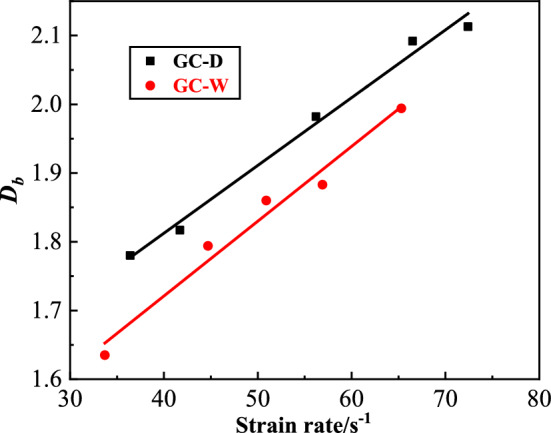
The fractal dimension of GC fragments.

The specimen fragments were sieved by standard sieves, and the mass of the retained fragments on each sieve was weighed, then the *ln[M(r)/M*_*T*_*]-lnr* curves of GC were drawn, as shown in Fig. 13. From this figure, the datas have good linear correlation in the double logarithmic coordinates, indicating that the fragment size distribution has fractal characteristics. The fractal dimension of GC fragments is shown in Fig. 14. From this figure, the fractal dimension of GC fragments before water saturation is 1.780 ~ 2.113, and that after water saturation is 1.635 ~ 1.994. With the increase of strain rate, the fractal dimension of fragments increases. The larger the impact velocity, the greater the degree of damage. And the strain rate is positively correlated with the impact velocity. Therefore, with the increase of strain rate, the damage degree and internal damage of concrete increase, and the fractal dimension of fragments increases. With the same strain rate, the fractal dimension of GC after water saturation is smaller than that before water saturation, which is due to the viscous resistance effect of free water. The free water reduces the cracking degree of GC after water saturation.

## Dynamic constitutive model

### Idea of model construction

Firstly, according to Weibull statistical damage distribution theory, a static damage constitutive model for GC is established. Then the static damage constitutive model is modified by introducing strain rate strengthening factor and water saturation influence factor, and the dynamic constitutive model of GC after water saturation is obtained. Finally, the model parameters are calibrated and the accuracy of the model is verified according to the existing test results.

### Static damage constitutive model

Based on the statistical damage theory, the static damage constitutive model of GC is established^35^. The damage variable (*D*) is defined based on the change of elastic modulus, as shown in Eq. (7).

7 $$ D = 1 - \frac{{E_{f} }}{E} $$

where $E$ and $E_{f}$ are the initial and effective elastic modulus, respectively. And the damage constitutive model of materials can be expressed as Eq. (8) based on the linear damage mechanics theory ^36^.

8 $$ \sigma = E\left( {1 - D} \right)\varepsilon $$

It is assumed that GC is composed of a large number of micro-units. On the one hand, these micro-units are large enough to contain many damage defects. On the other hand, these micro-units are small enough to be treated as particles compared to the whole concrete structure. Because the distribution of damage defects in concrete is random, it can be considered that the strength distribution of these micro-elements is random. The existing researches have shown that the randomness of the micro-unit strength distribution of the above materials can usually be characterized by some statistical laws, such as Weibull distribution, normal distribution, power function distribution, etc.^37,38^. And for concrete, its strength distribution of micro-units usually conforms to Weibull distribution^39^.

Therefore, in order to combine the damage theory and statistical theory to construct the corresponding statistical damage constitutive model, this study makes the following assumptions.

1. The strength distribution of micro-units of GC follows two-parameter Weibull distribution, and its probability density distribution function is Eq. (9).
  9 $$ P\left( F \right) = \frac{m}{{F_{0} }}\left( {\frac{F}{{F_{0} }}} \right)^{m - 1} e^{{ - \left( {\frac{F}{{F_{0} }}} \right)^{m} }} $$
  where *F* is the strength variable of micro-units, $m$ and $F_{0}$ are Weibull distribution parameters.
2. The damage evolution of geopolymeric concrete under loading can be regarded as a continuous process of micro-units cumulative failure. The decrease of the elastic modulus of GC at damaged state can be expressed as the failure of a certain number of micro-units. So the damage variable (*D*) can be expressed as Eq. (10).
  10 $$ D = N_{f} /N $$
  where the *N* and *N*_*f*_ are the number of all micro-units and destroyed micro-units.

Based on the above assumption, when the load increases from 0 to* F*, the *N*_*f*_ is shown in Eq. (11).

11 $$ N_{f} = \mathop \int \limits_{0}^{F} NP\left( F \right)dF = N\left( {1 - e^{{ - \left( {\frac{F}{{F_{0} }}} \right)^{m} }} } \right) $$

Equation (10) is substituted to Eqs. (11), and (12) is obtained.

12 $$ D = 1 - e^{{ - \left( {\frac{F}{{F_{0} }}} \right)^{m} }} $$

Equation (12) is substituted to Eq. (8), and the static damage constitutive model of GC can be obtained, as shown in Eq. (13).

13 $$ \sigma = E\varepsilon e^{{ - \left( {\frac{F}{{F_{0} }}} \right)^{m} }} $$

The strength (*F*) of micro-units in this model has different expressions under different strength criterion. Based on the Mohr–Coulomb strength criterion, *F* can be expressed as Eq. (14) ^40^.

14 $$ F = E\varepsilon \left( {1 + \sin \varphi } \right) $$

where the $\varphi$ is the internal friction angle.

### Dynamic constitutive model

#### Modification of static damage constitutive model

Strain rate and water saturation have significant effects on the stress (*σ*) of GC. Therefore, the static damage constitutive model is modified by introducing strain rate strengthening factor and water saturation influence factor.

1. Strain rate strengthening factor
  The strain rate strengthening factor ($R_{sr}$) is introduced on the basis of Eq. (13), as shown in Eq. (15).
  15 $$ \sigma = R_{sr} E\varepsilon e^{{ - \left( {\frac{F}{{F_{0} }}} \right)^{m} }} $$
  In the existing researches, $R_{{{\text{sr}}}}$ is usually defined as an increasing function of strain rate to reflect the strengthening effect of strain rate. For example, in the HJC constitutive model, the strain rate strengthening factor is defined as $R_{{{\text{sr}}}} = 1 + C_{0} ln\varepsilon^{\prime *}$, where $C_{0}$ is the strain rate coefficient and $\varepsilon^{\prime *}$ is the dimensionless strain rate. In this study, $R_{{{\text{sr}}}}$ is defined as Eq. (16).
  16 $$ R_{{{\text{sr}}}} = B_{1} + B_{2} \log \left( {\varepsilon^{\prime}} \right) + B_{3} \left[ {\log \left( {\varepsilon^{\prime}} \right)} \right]^{2} $$
  where $\varepsilon^{\prime}$ is strain rate, $B_{1}$, $B_{2}$ and $B_{3}$ are model parameters.
2. Water saturation influence factor.
  On the basis of introducing the strain rate strengthening factor, the swater influence factor ($R_{w}$) is further introduced, as shown in Eq. (17).
  17 $$ \sigma = R_{sr} R_{w} E\varepsilon e^{{ - \left( {\frac{F}{{F_{0} }}} \right)^{m} }} $$
  $R_{w}$ is defined as a function of the strain rate ($\varepsilon^{\prime}$), as shown in Eq. (18).
  18 $$ R_{w} = B_{4} + B_{5} \log \left( {\varepsilon^{\prime}} \right) $$
  where $B_{4}$ and $B_{5}$ are model parameters.

In addition, in the process of calibrating the model parameters, it is found that the distribution parameters (*m*, $F_{0}$) have great influence on the shape of the stress–strain curve, and their values are different at different strain rates. Therefore, in order to improve the accuracy of the model and facilitate parameters fitting, the distribution parameters (*m*, $F_{0}$) are defined as functions of the strain rate ($\varepsilon^{\prime}$), respectively, as shown in Eq. (19).

19 $$ \left\{ {\begin{array}{*{20}c} {m = H_{1} + H_{2} \log \left( {\varepsilon^{\prime}} \right) + H_{3} \left[ {\log \left( {\varepsilon^{\prime}} \right)} \right]^{2} } \\ {F_{0} = H_{4} + H_{5} \log \left( {\varepsilon^{\prime}} \right) + H_{6} \left[ {\log \left( {\varepsilon^{\prime}} \right)} \right]^{2} } \\ \end{array} } \right. $$

where ${H}_{1}$~${H}_{6}$ are model parameters.

Finally, through the corresponding modification, the dynamic constitutive model of GC after water saturation can be obtained, as shown in Eq. (20).

20 $$ \sigma = \left\{ {B_{1} + B_{2} \log \left( {\varepsilon^{\prime}} \right) + B_{3} \left[ {\log \left( {\varepsilon^{\prime}} \right)} \right]^{2} } \right\}\left[ {B_{4} + B_{5} \log \left( {\varepsilon^{\prime}} \right)} \right]E\varepsilon e^{{ - \left( {\frac{F}{{F_{0} }}} \right)^{m} }} $$

where $$F = E\varepsilon \left( {1 + \sin \varphi } \right)$$.

#### Parameters calibration

According to Eq. (19), the dynamic constitutive model of GC after water saturation contains the following parameters, *B*_*1*_ ~ *B*_*5*_, *H*_*1*_ ~ *H*_*6*_, *E* and *φ*. The specific determination method of them is as follows.

1. The secant modulus is taken as elastic modulus (*E*), namely *E *= (*σ*_*0.6*_ − *σ*_*0.4*_)/(*ε*_*0.6*_ − *ε*_*0.4*_). Where, *σ*_*0.6*_=0.6*f*, *σ*_*0.4*_=0.4*f*, and *f* is the peak stress. *ε*_*0.6*_ and *ε*_*0.4*_ are the corresponding strains when the stress is *σ*_*0.6*_ and *σ*_*0.4*_ on the rising section of the stress-strain curve, respectively.
2. According to the peak stress of GC-D and GC-W in Fig. 8, the values of *B*_*1*_ ~ *B*_*5*_ can be obtained by regression analysis. The values of *B*_*1*_ ~ *B*_*5*_ after regression analysis are shown in Table 2. It should be noted that when Eq. (20) is used for GC-D, *B*_*4*_ is 1 and *B*_*5*_ is 0, that is, $R_{w}$ is 1.

**Table 2:** The values of *B*_*1*_ ~ *B*_*5*_.

B1	B2	B3	B4	B5
− 5.225	7.212	− 1.898	0.784	0.164
3. *m* and $F_{0}$ are repeatedly calibrated according to the experimental stress–strain curves, and then the values of *H*_*1*_ ~ *H*_*6*_ are obtained by regression analysis, as shown in Table 3.

**Table 3:** The values of *H*_*1*_ ~ *H*_*6*_.

	H1	H2	H3	H4	H5	H6
GC-D	− 124.445	149.225	− 42.489	0.009	0.007	− 0.002
GC-W	− 330.360	412.119	− 125.834	0.163	− 0.192	0.061
4. *φ* is obtained by further adjusting and calibrating according to the experimental stress–strain curves based on the existing empirical value^31^. According to the research results in Reference^35^, the value of *φ* has little effect on the fitting results of the model, so this parameter determination method will not have a great influence on the accuracy of the model.

#### Model verification

**Figure 15:**
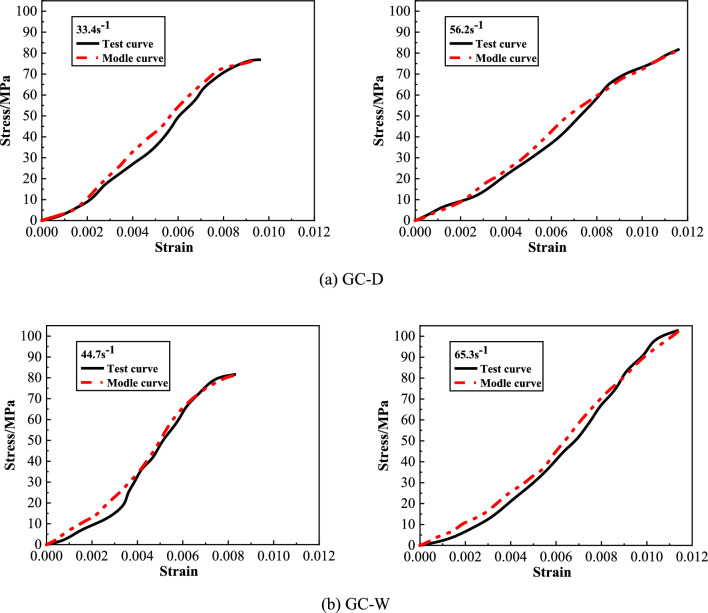
Comparison between model fitting curves and experimental curves.

In order to verify the accuracy of the model, the model fitting curves and experimental stress–strain curves of GC-D and GC-W are shown in Fig. 15. From this figure, in general, the model fitting curves is in good agreement with the experimental stress–strain curves, and the characteristic strength and strain are close to each other, which shows that the modeling method adopted in this study is feasible and the dynamic constitutive model can accurately reflect the impact mechanical behavior of GC after water saturation. The dynamic constitutive model in this study has certain engineering practical value for the design calculation, stress analysis and safety evaluation of underwater GC structure. Meanwhile, it can also provide some reference for the corresponding numerical simulation.

## Conclusion

The dynamic compressive test of geopolymer concrete (GC) before and after water saturation was carried out, and the effects of water saturation and strain rate on the impact toughness and fracture morphology of GC were studied. Based on Weibull statistical damage distribution theory, the dynamic constitutive model of GC after water saturation was constructed. The main conclusions are as follows.

1. The dynamic peak strain and specific energy absorption of GC before or after water saturation have strain rate strengthening effect. The impact toughness of GC decreases after water saturation.
2. The dynamic peak strain and specific energy absorption of GC decrease after water saturation, and the maximum extent of the decrease are 26.16 and 13.90%, respectively.
3. The size distribution of GC fragments before or after water saturation has fractal characteristics. The fractal dimension of GC fragments after water saturation is smaller than that before water saturation.
4. The modeling method based on Weibull statistical damage distribution theory is feasible. The dynamic constitutive model fitting curves are in good agreement with the experimental stress–strain curves.

## Data Availability

The data that support the findings of this study are available on request from the corresponding author.
